# Risk Assessment by Presurgical Tractography Using Navigated TMS Maps in Patients with Highly Motor- or Language-Eloquent Brain Tumors

**DOI:** 10.3390/cancers12051264

**Published:** 2020-05-17

**Authors:** Nico Sollmann, Haosu Zhang, Alessia Fratini, Noémie Wildschuetz, Sebastian Ille, Axel Schröder, Claus Zimmer, Bernhard Meyer, Sandro M. Krieg

**Affiliations:** 1 Department of Neurosurgery, Klinikum rechts der Isar, Technische Universität München, Ismaninger Str. 22, 81675 Munich, Germany; Nico.Sollmann@tum.de (N.S.); Haosu.Zhang@gmail.com (H.Z.); Alessia.Fratini95@gmail.com (A.F.); Noemie.Wildschutz@hotmail.com (N.W.); Sebastian.Ille@tum.de (S.I.); Axel.Schroeder@tum.de (A.S.); Bernhard.Meyer@tum.de (B.M.); 2 TUM-Neuroimaging Center, Klinikum rechts der Isar, Technische Universität München, 81675 Munich, Germany; 3 Department of Diagnostic and Interventional Neuroradiology, Klinikum rechts der Isar, Technische Universität München, Ismaninger Str. 22, 81675 Munich, Germany; Claus.Zimmer@tum.de

**Keywords:** arcuate fascicle, brain tumor, corticospinal tract, diffusion tensor imaging, navigated transcranial magnetic stimulation, risk assessment

## Abstract

Patients with functionally eloquent brain lesions are at risk of functional decline in the course of resection. Given tumor-related plastic reshaping and reallocation of function, individual data are needed for patient counseling and risk assessment prior to surgery. This study evaluates the utility of mapping by navigated transcranial magnetic stimulation (nTMS) and nTMS-based diffusion tensor imaging fiber tracking (DTI FT) for individual risk evaluation of surgery-related decline of motor or language function in the clinical setting. In total, 250 preoperative nTMS mappings (100 language and 150 motor mappings) derived from 216 patients (mean age: 57.0 ± 15.5 years, 58.8% males; glioma World Health Organization (WHO) grade I & II: 4.2%, glioma WHO grade III & IV: 83.4%, arteriovenous malformations: 1.9%, cavernoma: 2.3%, metastasis: 8.2%) were included. Deterministic tractography based on nTMS motor or language maps as seed regions was performed with 25%, 50%, and 75% of the individual fractional anisotropy threshold (FAT). Lesion-to-tract distances (LTDs) were measured between the tumor mass and the corticospinal tract (CST), arcuate fascicle (AF), or other closest language-related tracts. LTDs were compared between patients and correlated to the functional status (no/transient/permanent surgery-related paresis or aphasia). Significant differences were found between patients with no or transient surgery-related deficits and patients with permanent surgery-related deficits regarding LTDs in relation to the CST (*p* < 0.0001), AF (*p* ≤ 0.0491), or other closest language-related tracts (*p* ≤ 0.0435). The cut-off values for surgery-related paresis or aphasia were ≤12 mm (LTD—CST) and ≤16 mm (LTD—AF) or ≤25 mm (LTD—other closest language-related tract), respectively. Moreover, there were significant associations between the status of surgery-related deficits and the LTD when considering the CST (range r: −0.3994 to −0.3910, *p* < 0.0001) or AF (range r: −0.2918 to −0.2592, *p* = 0.0135 and *p* = 0.0473 for 25% and 50% FAT). In conclusion, this is the largest study evaluating the application of both preoperative functional mapping and function-based tractography for motor and language function for risk stratification in patients with functionally eloquent tumors. The LTD may qualify as a viable marker that can be seamlessly assessed in the clinical neurooncological setup.

## 1. Introduction

Patients who present with brain lesions affecting functionally eloquent brain structures on a cortical and/or subcortical level put the neurosurgeon at a particular challenge as removal could lead to unintended decline of motor or language function [1,2,3,4]. Importantly, tumor-induced plastic reshaping, involving spatial reallocation of function within in the brain, can have huge effects and causes highly individual functional anatomy that can be very divergent from normal brains of healthy subjects [5,6,7,8]. As a consequence, individual functional anatomy should be considered to minimize the risk of functional worsening and to better counsel patients prior to tumor resection.

The state-of-the-art preoperative neurosurgical workup in patients with functionally eloquent lesions includes functional mapping techniques to identify and spatially enclose eloquent structures [9,10,11]. A technique that gains increasing attention is navigated transcranial magnetic stimulation (nTMS), which has become available for preoperative functional mapping in the 2000s [12,13,14]. Since then, nTMS has mostly been used for motor or language mappings in the routine clinical setting as it has shown good accordance with intraoperative direct electrical stimulation (DES) as the gold-standard method, which holds particularly true for mapping of the motor cortex [12,15,16]. Further, it is suggested that application of preoperative nTMS mapping facilitates improved clinical outcome as it has demonstrated to increase the extent of resection, while keeping perioperative functional deterioration on a comparatively low level [17,18,19,20,21]. Lately, nTMS mapping has been combined with diffusion tensor imaging fiber tracking (DTI FT) to enable delineation of white matter pathways such as the corticospinal tract (CST) or arcuate fascicle (AF) based on functional data [22,23,24].

However, the role of nTMS and nTMS-based DTI FT for risk stratification is just emerging. To date, only few studies have investigated the potential for risk assessment, which could allow nTMS developing into an even more effective and multifarious preoperative tool beyond mere purposes during preoperative planning and resection guidance. Specifically, recent studies used nTMS-based DTI FT to measure the lesion-to-tract distance (LTD) for the CST or AF and other closest language-related tracts and to determine minimum LTDs as cut-off values to prevent perioperative functional deterioration, suggesting that a minimum LTD of approximately 1 cm might be considered as a warning criteria for functional worsening [25,26,27]. Another study used nTMS-based DTI FT to track transcallosal fibers, demonstrating that the detectability of such fibers based on nTMS data may be considered as a risk factor for surgery-related aphasia with a specificity of up to 93% [28]. Furthermore, the resting motor threshold (rMT) has been related to risk assessment, with studies reporting on negative correlations between the rMT and LTDs for the CST among patients with surgery-related paresis, and on new deficits being associated with a pathological excitability of the motor cortices as expressed by an interhemispheric rMT ratio [25,26].

Despite increasing interest in the role of function-based DTI FT for risk assessment, further investigation in large cohorts is needed to expand recent work. In particular, the limited body of research on the matter has focused on patients with motor-eloquent tumors and high-grade glioma, leaving it questionable whether patients with language-eloquent lesions or low-grade glioma may also profit from nTMS-based risk stratification. Furthermore, potential direct associations between LTDs and surgery-related deficits have not yet been evaluated in detail. Thus, the aim of this study is to further refine the role of nTMS and nTMS-based DTI FT for preoperative risk assessment.

## 2. Methods

### 2.1. Ethics

This study was approved by the local institutional review board (ethics committee registration numbers: 2793/10, 5811/13, 223/14, and 336/17) and was performed in accordance with the Declaration of Helsinki. Written informed consent was obtained from all patients.

### 2.2. Study Design and Patient Inclusion

The present study uses monocentric and prospectively collected data of patients with motor- or language-eloquent tumors who were treated at our university hospital. All patients enrolled underwent preoperative nTMS motor or language mapping as well as nTMS-based DTI FT prior to tumor resection or biopsy between July 2013 and January 2020. Digital patient charts were accessible for collection of patient- and tumor-related information, including tumor entity, grading according to the World Health Organization (WHO), and status of mutation of the isocitrate dehydrogenase (IDH) in glioma patients as determined by histopathological evaluation. Small subcohorts of the present investigation have been investigated previously [26,27].

The following inclusion criteria were defined:

- Written informed consent;
- Age above 18 years;
- Suspected right- or left-hemispheric motor-eloquent and/or left-hemispheric language-eloquent tumor location according to initial anatomical magnetic resonance imaging (MRI; suggesting infiltration or compression of anatomically suspected cortical motor-eloquent or language-eloquent areas and/or suspected close proximity to the CST or subcortical language-related pathways);
- Availability of preoperative 3-Tesla MRI including diffusion tensor imaging (DTI) with 32 diffusion directions;
- Clinical indication for preoperative nTMS language or motor mapping and nTMS-based DTI FT; surgery for tumor resection or biopsy;
- Regular preoperative, postoperative, and follow-up (FU) examinations (at least until the three-month FU visit) including respective assessments of motor or language function.

Furthermore, the following exclusion criteria were defined:

- Pregnancy;
- Implanted devices (e.g., deep brain stimulation electrodes or cochlear implants);
- Preoperative plegia and/or severe aphasia making motor or language mapping by nTMS impossible;
- Infratentorial tumor location;
- Relevant postoperative bleeding according to T2*- or susceptibility-weighted MRI with suspected affection of the motor cortex or course of the CST and/or language cortex or course of major subcortical language-related pathways.

### 2.3. Magnetic Resonance Imaging

Cranial MRI was performed within the week before nTMS mapping on a 3-Tesla scanner (Achieva, Achieva dStream, or Ingenia; Philips Healthcare, Best, The Netherlands) using a 32-channel head coil. The standardized imaging protocol for brain tumors at our hospital includes, among further dedicated sequences, a three-dimensional (3D) fluid attenuated inversion recovery (FLAIR) sequence (repetition time (TR)/echo time (TE): 4800/277 ms, 1 mm^3^ isovoxel covering the whole head), a DTI sequence (TR/TE: 5000/78 ms, voxel size: 2 × 2 × 2 mm^3^, 32 diffusion gradient directions), and a 3D gradient echo sequence (TR/TE: 9/4 ms, 1 mm^3^ isovoxel covering the whole head) without and with application of an intravenous contrast agent.

Postoperative MRI was carried out within the first 48 h after surgery using the same sequences, explicitly also including T2*- or susceptibility-weighted and diffusion-weighted MRI to assess presence of bleeding or perioperative ischemia, respectively. Imaging for FU examination was scheduled in fixed intervals depending on the tumor entity and clinical course of the patients, again using the same imaging protocol.

### 2.4. Definition of Functional Deficits

Patient examination during the preoperative, postoperative, and three-month FU setting consisted of standardized assessments of sensory function, coordination, cranial nerve function, muscle strength according to the British Medical Research Council (BMRC) scale, and language function according to the Aachen Aphasia Test with categorization of the language status into four grades.

Regarding motor strength, no paresis was present when motor strength was 5/5 for all extremities according to the BMRC scale, while a paresis was defined when motor strength was <5/5. Concerning language deficits, we discriminated between no deficit (grade 0), mild deficit (grade 1; normal speech comprehension and/or conversational speech with slight amnesic aphasia, adequate communication ability), medium deficit (grade 2; minor disruption of speech comprehension and/or conversational speech, adequate communication ability), and severe deficit (grade 3; major disruption of speech comprehension and/or conversational speech, clear impairment of communication ability) [19,21,27]. Evaluations were compared between the preoperative and postoperative as well as preoperative and three-month FU examinations. Furthermore, for new or aggravated perioperative functional deficits, two categories were established [26,27]:

- Transient paresis/transient aphasia: any new or increased motor or language deficit owing to surgery that resolved within the regular FU interval;
- Permanent paresis/permanent aphasia: any new or increased motor or language deficit owing to surgery that did not resolve to the preoperative status within the regular FU interval.

### 2.5. Mapping by Navigated Transcranial Magnetic Stimulation

Preoperative motor or language mapping was performed with a nTMS system capable of electric-field navigation (NBS system 4.3 or 5.0; Nexstim Plc., Helsinki, Finland). Mappings were conducted according to a standardized protocol using a focal figure-of-eight coil with biphasic pulse wave application [14,29,30]. Both motor and language mapping by nTMS started with the determination of the rMT using the built-in threshold hunting algorithm [29,30].

#### 2.5.1. Motor Mapping

Motor mapping of the tumor-affected hemisphere used electromyography recordings of motor evoked potentials (MEPs) from the contralateral side considering the abductor pollicis brevis, abductor digiti minimi, flexor carpi radialis, and biceps brachii, tibialis anterior, and gastrocnemius muscles. During mapping of cortical representations of the upper extremity, an intensity of 105–110% of the individual rMT was used, whereas an intensity of at least 130% of the rMT was applied during stimulation of representations of lower extremity muscles [29]. Post-hoc analysis was performed to identify motor-positive nTMS points, which were defined as stimulation spots that showed an amplitude of MEPs larger or equal to 50 µV with onset latencies within the typical ranges [26,29].

#### 2.5.2. Language Mapping

Language mapping of the tumor-affected hemisphere used a standardized object-naming task and started with at least two rounds of initial baseline trials. An intensity of 100% of the individual rMT was used during repetitive nTMS with 5 Hz/5 pulses, targeting 46 predefined cortical spots that were stimulated six times in total [29]. Post-hoc analysis used video recordings of the language mapping sessions to identify language-positive nTMS points, which were defined as stimulation spots that led to naming errors during task performance of one of the following categories: no responses, performance errors, neologisms, phonological paraphasia, and semantic paraphasia [27,29,31].

### 2.6. Tractography Based on Navigated Transcranial Magnetic Stimulation

Preoperative nTMS-based DTI FT was performed using a deterministic tracking algorithm implemented in the neurosurgical neuronavigation environment (Brainlab Elements version 3.1.0; Brainlab AG, Munich, Germany). Tractography was based on the motor- or language-positive nTMS points used for seeding, which were fused with the preoperative FLAIR, DTI, and contrast-enhanced 3D gradient echo sequences [21,29,30]. Eddy current correction as implemented in the tractography application was used throughout for DTI [21,29,30].

#### 2.6.1. Tracking of the Corticospinal Tract

**Figure 1:**
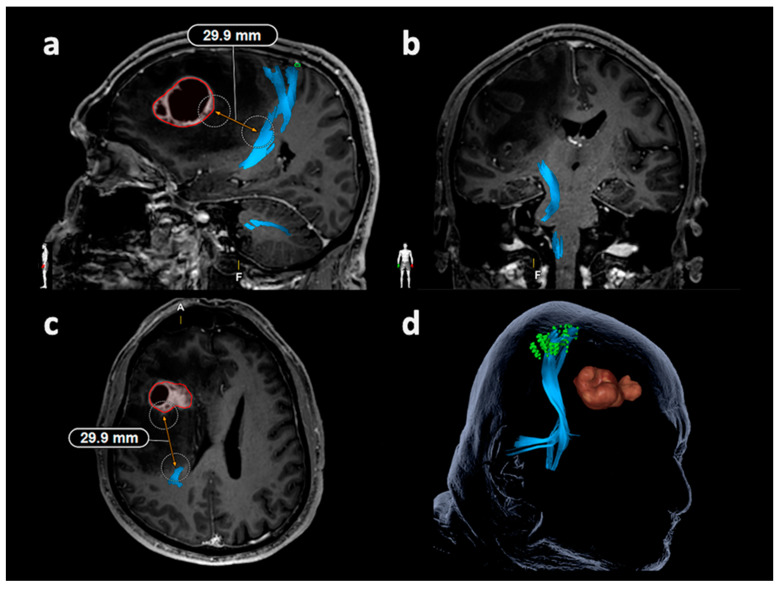
Measurement of the lesion-to-tract distance (LTD) in relation to the corticospinal tract (CST). This illustrative patient case depicts the LTD measurement in relation to the CST using diffusion tensor imaging fiber tracking (DTI FT) based on the motor map derived from navigated transcranial magnetic stimulation (nTMS). The CST is shown in blue, the motor map is depicted in green, and the tumor volume is enclosed in red. During LTD measurements, all planes were considered ((**a**) sagittal plane, (**b**): coronal plane, (**c**): axial plane), with the LTD measurement of 29.9 mm being shown in parts (**a**) and (**c**) in this patient case. The three-dimensional (3D) head model including the structures of interest is depicted in part (**d**).

Tractography of the CST for the tumor-affected hemisphere used the motor-positive nTMS points with a rim of 2 mm, which was added to each individual point, combined with a manually drawn region of interest (ROI) at the level of the ipsilateral brainstem [22,26,30]. Tracking of fibers connecting or passing through these ROIs, thus visualizing the CST, was performed with a minimum fiber length (FL) of 100 mm and a fractional anisotropy (FA) value corresponding to 25%, 50%, and 75% of the individual FA threshold (FAT) [24,26,30]. The LTD for the CST was defined in FLAIR and/or contrast-enhanced 3D gradient echo sequences of all geometric planes by linearly measuring the minimum distance between the border of the solid tumor mass and the closest CST fibers (Figure 1) [26]. Perifocal diffuse infiltration zones or edema were not taken into account for LTD measurements.

#### 2.6.2. Tracking of the Arcuate Fascicle and Other Language-Related Fiber Tracts

**Figure 2:**
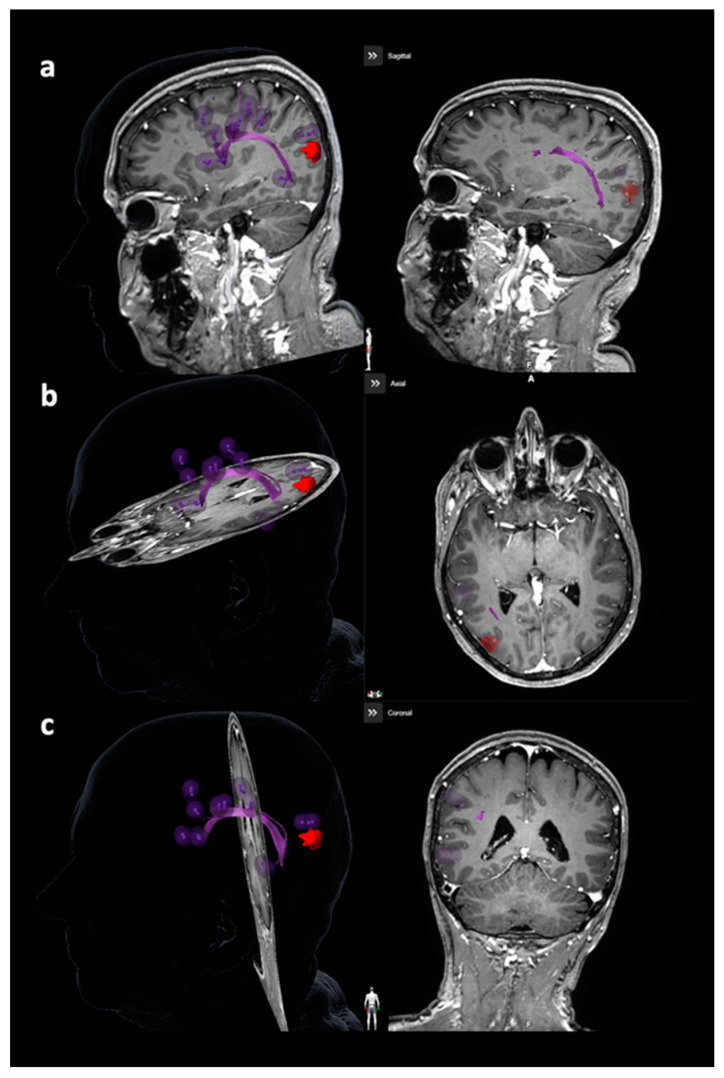
Visualization of the arcuate fascicle (AF). This figure depicts the AF in purple considering all planes ((**a**): sagittal plane, (**b**): axial plane, (**c**): coronal plane), with the reconstruction being purely based on diffusion tensor imaging fiber tracking (DTI FT) using the language map derived from navigated transcranial magnetic stimulation (nTMS). The language map is represented by purple spots, while the tumor volume is enclosed in red.

**Figure 3:**
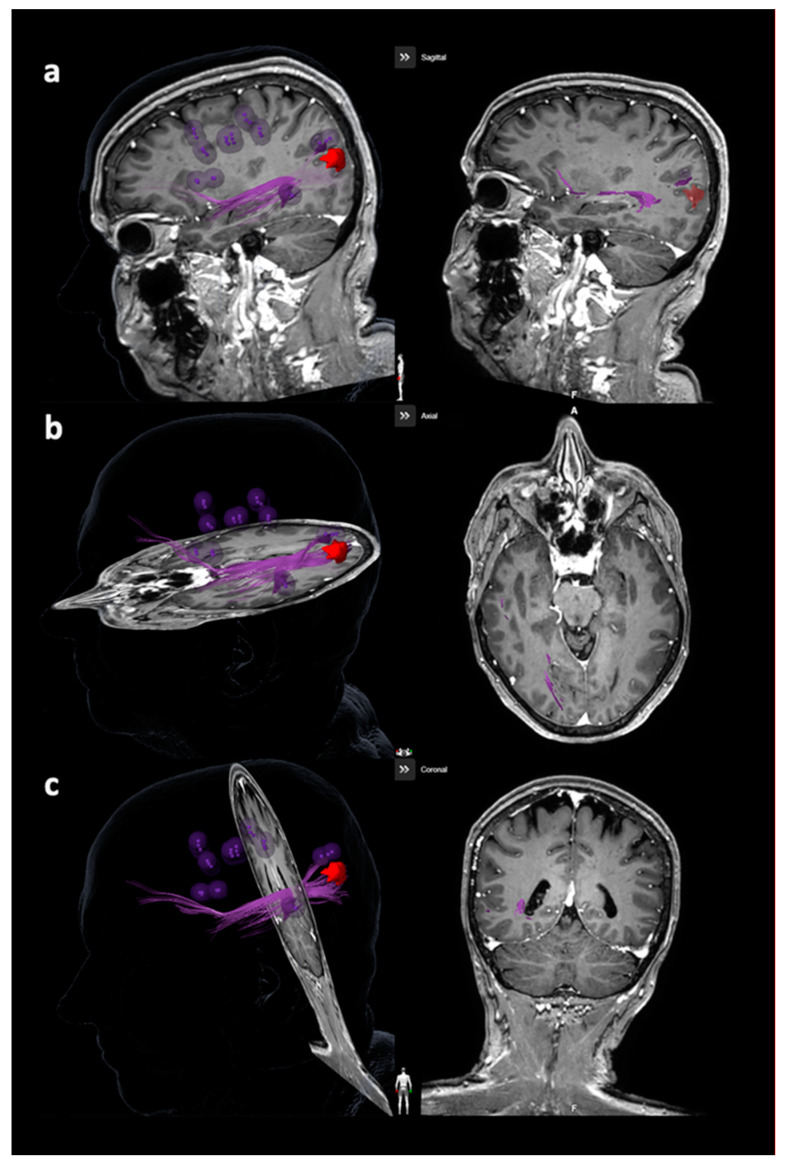
Visualization of the frontooccipital fascicle (FoF). This figure depicts the FoF in purple considering all planes ((**a**): sagittal plane, (**b**): axial plane, (**c**): coronal plane), with the reconstruction being purely based on diffusion tensor imaging fiber tracking (DTI FT) using the language map derived from navigated transcranial magnetic stimulation (nTMS). The language map is represented by purple spots, while the tumor volume is enclosed in red.

**Figure 4:**
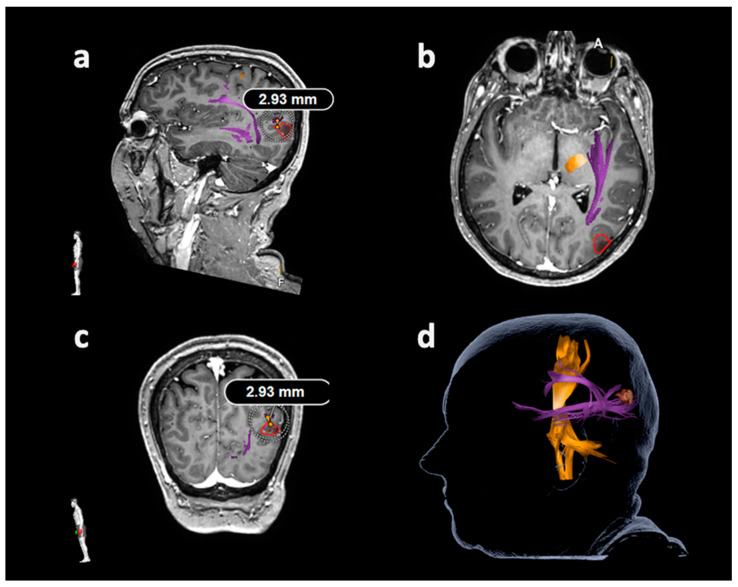
Measurement of the lesion-to-tract distance (LTD) in relation to the frontooccipital fascicle (FoF). This illustrative patient case illustrates the LTD measurement in relation to the FoF as the closest language-related tract except for the arcuate fascicle (AF), applying diffusion tensor imaging fiber tracking (DTI FT) based on the language map derived from navigated transcranial magnetic stimulation (nTMS). The FoF and AF are depicted in purple, the corticospinal tract is shown in orange, and the tumor volume is enclosed in red. During LTD measurements, all planes were considered ((**a**): sagittal plane, (**b**): axial plane, (**c**): coronal plane), with the LTD measurement of 2.93 mm being shown in parts (**a**) and (**c**) in this patient case. The three-dimensional (3D) head model including the structures of interest is provided in part (**d**).

Tractography of the AF or other closest language-related tracts considering the superior longitudinal fascicle (SLF), inferior longitudinal fascicle (ILF), uncinate fascicle (UC), and frontooccipital fascicle (FoF) was performed with language-positive nTMS points with a rim of 5 mm as the only ROI (Figure 2 and Figure 3) [23,27,30]. Similar to nTMS-based DTI FT of the CST, a minimum FL of 100 mm was combined with 25%, 50%, and 75% FAT [23,27,30]. The LTD for the AF or other closest language-related tract (either SLF, ILF, UC, or FoF) was defined in FLAIR and/or contrast-enhanced 3D gradient echo sequences by linearly measuring the minimum distance between the border of the solid tumor mass and the closest fibers of the AF or other closest language-related tracts (Figure 4) [27].

### 2.7. Statistics

GraphPad Prism (version 7.0; GraphPad Software Inc., La Jolla, CA, USA) was used for statistical analyses. A *p*-value <0.05 was considered statistically significant.

Descriptive statistics were calculated for patient, mapping, and tractography characteristics as well as measured LTDs considering the CST, AF, and other closest language-related tracts (using nTMS-based DTI FT with 25%, 50%, and 75% FAT for tractography). For patients harboring glioma, the LTDs were compared between patients showing IDH-wildtype and patients presenting IDH-mutant status using Mann–Whitney U tests. The rMT was compared between the tumor-affected and contralateral hemisphere (in case that bihemispheric rMT determination was achieved) using Wilcoxon matched-pairs signed rank tests. On the basis of ranges for LTDs, cut-off values for surgery-related permanent deficits were determined, with the cut-off value being defined as the maximum measured LTD that was observed among all patients with surgery-related permanent deficits. Consequently, patients who showed distances above the respective cut-off values for the CST, AF, or other closest language-related tracts did not suffer from surgery-related permanent deficits.

The LTDs were compared between patients presenting no or transient and permanent surgery-related deficits, which was achieved separately for the three different tracking adjustments and for the CST, AF, or other closest language-related tracts, respectively, using Mann–Whitney U tests for group comparisons. Furthermore, for patients with permanent surgery-related deficits, discrimination was made between the whole group and the subgroup of patients who showed permanent deficits without signs of perioperative ischemia according to postoperative diffusion-weighted MRI. Correlations between the status of surgery-related deficits (no or transient and permanent surgery-related deficits) and LTDs were assessed by Spearman correlation coefficients.

## 3. Results

### 3.1. Patients

This study included 250 preoperative nTMS mappings (100 language and 150 motor mappings) derived from 216 patients with motor- and/or language-eloquent location of an intracranial space-occupying lesion (Table 1). Twenty-nine patients underwent both motor and language mapping by nTMS during the same session (owing to tumor location suspected to affect both motor and language function), and five patients were mapped two times at different time points owing to tumor relapse with a second scheduled surgery for re-resection.

### 3.2. Mapping and Tractography

Motor and/or language mapping of the tumor-affected hemisphere was possible in all patients without technical difficulties or premature termination of the mapping session (e.g., owing to discomfort or exhaustion of the patient; Table 2). No adverse events were observed in the course of stimulations. Furthermore, tractography of the CST, AF, and/or other closest language-related tract was achieved in all patients using nTMS data (Table 2). The measurements of LTDs took approximately 5 to 10 min per patient.

### 3.3. Lesion-To-Tract Distances

#### 3.3.1. Corticospinal Tract

In all patients with nTMS motor mappings together, the lowest LTDs for measurements in relation to the CST were observed for tracking with 25% FAT (mean LTD ± SD: 8.2 ± 9.4 mm, range: 0.0–42.4 mm). There were no statistically significant differences in terms of LTDs when comparing patients diagnosed with IDH-wildtype gliomas with patients with IDH-mutant gliomas (*p* > 0.05).

A statistically significant difference was found between patients with no or transient surgery-related paresis and patients with permanent surgery-related paresis for nTMS-based DTI FT using 25%, 50%, and 75% FAT, respectively (*p* < 0.0001 each; Table 3). When excluding patients with perioperative ischemia, statistical significance was preserved throughout (*p* < 0.0001 each; Table 3).

Patients with no or transient surgery-related paresis presented with clearly higher LTDs for the CST (Table 3). The cut-off value for surgery-related permanent paresis was 11.8 mm; thus, no patient with an LTD above this value showed permanent functional decline of motor function (Table 3).

#### 3.3.2. Arcuate Fascicle and Other Language-Related Fiber Tracts

Among the patients with nTMS language mappings, the lowest LTDs for measurements in relation to the AF as well as the other closest language-related tracts were obtained for tracking with 25% FAT (AF: mean LTD ± SD: 10.7 ± 11.3 mm, range: 0.0–41.1 mm; other closest language-related fiber tracts except for the AF: mean LTD ± SD: 8.8 ± 12.1 mm, range: 0.0–53.0 mm). There were no statistically significant differences revealed for LTDs when comparing patients harboring IDH-wildtype gliomas with patients with IDH-mutant gliomas (*p* > 0.05).

Statistically significant differences were revealed between patients with no or transient surgery-related aphasia and patients with permanent surgery-related aphasia for nTMS-based tractography, which was observed for the AF as well as other closest language-related fiber tracts and the three different tracking adjustments, respectively (AF: 25% FAT: *p* = 0.0005, 50% FAT: *p* = 0.0012, 75% FAT: *p* = 0.0491; other closest language-related tract: 25% FAT: 0.0113, 50% FAT: 0.0147, 75% FAT: 0.0435; Table 4). After exclusion of patients with perioperative ischemia, group differences remained statistically significant for the AF using 25% FAT for nTMS-based DTI FT (*p* = 0.0119; Table 4).

Similar to the results for nTMS-based DTI FT of the CST, patients with no or transient surgery-related paresis presented with considerably higher LTDs (Table 4). The cut-off value for surgery-related permanent aphasia was 15.4 mm regarding the AF and 24.1 mm concerning the other closest language-related tracts (Table 4).

### 3.4. Correlations between Lesion-To-Tract Distances and Deficits

For nTMS-based DTI FT of the CST, there was a statistically significant association between the status of surgery-related paresis and the LTD when using 25%, 50%, or 75% FAT for tracking, respectively (25% FAT: r = −0.3910, 50% FAT: r = −0.3923, 75% FAT: r = −0.3994, *p* < 0.0001 each; Table 5). Regarding the AF, statistically significant correlations were found for tracking with 25% FAT (r = −0.2918, *p* = 0.0135) and 50% FAT (r = −0.2662, *p* = 0.0473; Table 5). No statistically significant correlations were revealed for other closest language-related tracts regarding the status of surgery-related paresis and the LTD when excluding patients showing perioperative ischemia (*p* > 0.05 each; Table 5).

## 4. Discussion

### 4.1. General Considerations

This study evaluated preoperative risk assessment regarding functional outcome in patients harboring functionally eloquent brain tumors using nTMS mapping as well as nTMS-based DTI FT. In this regard, analyses were performed for all included patients together as well as separately for patients without evidence of perioperative ischemia according to postoperative diffusion-weighted MRI. The main findings are that (i) patients with no or transient surgery-related deficits show significantly higher LTDs when compared with patients with permanent surgery-related deficits (particularly for the CST; Table 3 and Table 4), (ii) there are respective cut-off values for surgery-related permanent deficits (11.8 mm for the CST, 15.4 mm for the AF, and 24.1 mm for the other closest language-related tracts; Table 3 and Table 4), and (iii) statistically significant correlations are present between the LTDs and the status of surgery-related deficits (CST and AF; Table 5).

It becomes increasingly evident that plasticity in the presence of tumor growth can cause considerable reallocation of functional anatomy [5,6,7,8]. Owing to highly individual functional representations, preoperative evaluation of the risk of functional decline seems indispensable. The approach and results of this study provide objective data that may be carefully considered in the preoperative clinical setup, probably not only supporting the neurosurgeon and the interdisciplinary tumor board in decision making, but also providing beneficial insights for other disciplines involved in later treatment, like radiation oncologists. Of note, seamless integration of nTMS maps and nTMS-based DTI FTI in the neurosurgical neuronavigation environment and use of such data for radiotherapy and radiosurgery planning have already been reported [30,32,33,34].

### 4.2. Lesion-To-Tract Distances for Motor Function

Regarding tractography of the CST, a limited body of previous research used functional data in the context of risk assessments in patients with brain tumors, mostly functional MRI [25,26,35,36,37,38]. Specifically, lesion-to-activation distances (LADs) were measured, with LADs of 10 mm or more leading to decreased risk profiles for postoperative functional decline [35,36,37,38]. Two recent studies comparable to the present investigation applied nTMS motor maps for seeding in risk evaluation, demonstrating that no new postoperative motor deficits occurred when the LTD was >8 mm (tracking with 75% FAT) and ≥12 mm regarding a new surgery-related permanent paresis (50% FAT and 75% FAT), respectively [25,26]. The LTD for the CST of this study is in good accordance with previous results, amounting to 11.8 mm when excluding patients with perioperative ischemia. For all patients together, the potential cut-off value was slightly higher and amounted to 13.4 mm. Thus, further evidence for the beneficial use of nTMS motor mapping and nTMS-based DTI FT of the CST for risk evaluation can be drawn from the present study.

### 4.3. Lesion-To-Tract Distances for Language Function

Analogously to the evaluation of the motor system, LTDs of the AF or other closest language-related tract were assessed. Few previous studies investigated associations of distances with language deficits, mainly using data other than nTMS language maps for seeding [27,38,39,40,41]. In a functional MRI-based study, the degree of SLF involvement was significantly different between preoperatively symptomatic and asymptomatic patients regarding functional impairment [38]. Furthermore, LTDs regarding the left-hemispheric SLF were correlated to the occurrence rate of postoperative language deficits, with the LTD threshold that best predicted occurrence amounting to 1 cm [41]. One previous study applying nTMS-based tractography for the AF revealed cut-off LTDs for permanent surgery-related aphasia of ≥ 8 mm (AF) and ≥ 11 mm (other closest language-related tract), respectively [27]. In the present study, slightly higher cut-off values of 15.4 mm for the AF and 24.1 mm for other closest language-related tracts were obtained when excluding patients who showed perioperative ischemia. Without exclusions, the potential cut-off values amounted to 27.1 mm and 24.1 mm, respectively. Discrepancies may be related to the lower case number and, related to that, comparatively low rates of permanent surgery-related deficits in the previous study, hampering statistical evaluations when considering subjects with new permanent deficits as a subgroup [27]. However, the previous study also revealed higher LTDs when considering other language-related tracts compared with the LTDs for the AF [27]. A similar trend is also revealed by the present study, but it has to be acknowledged that the human language network is very complex and not entirely understood, particularly in patients with brain tumors and functional reorganization, making it possible that incorporation of further tracts in addition to the SLF, ILF, UC, or FoF might be more accurate in future studies.

### 4.4. Correlations to Functional Outcome

Statistically significant associations between the LTDs and status of surgery-related deficits are shown in this study. Such correlation analyses have not been performed by previous studies using nTMS-based DTI FT for risk evaluations considering LTDs [25,26,27]. The higher the LTD, the lower the presence of surgery-related permanent deficits when considering the CST and AF. When not excluding patients with perioperative ischemia, similar results were also observed for the other closest language-related tracts. Evidently, the closer a tract is in relation to the site of surgery, the higher the risk for potential damage owing to the surgical procedure as well as to potential vascular affection. The finding that LTDs are highly different between patients with no or only transient surgery-related deficits and patients with permanent surgery-related deficits and that, in particular, correlations exist between LTDs and status of surgery-related functional decline, may enable the LTD to become a viable preoperative marker in the clinical setting.

### 4.5. Limitations and Perspectives

First, measurements of LTDs were derived from a cohort of patients with different entities of brain lesions, but most patients suffered from glioma. On the one hand, the results may then be applicable to consecutive patients in the clinical setting presenting with different entities. On the other hand, restriction to a particular histology may have enabled the determination of more specific cut-off values on the basis of growth or infiltration patterns, if any, that are variable between entities. Second, particularly the cortico-subcortial language network is highly complex, making it possible that measurements to other closest language-related tracts than the AF may not necessarily be in close relation to distinct postoperative aphasia grades. Furthermore, a potentially important role of the contralesional hemisphere has to be acknowledged as well, which has not been considered in investigations of the present study. Of note, previous work using nTMS mapping and/or nTMS-based tractography, particularly for exploration of the language network, has repeatedly suggested interhemispheric compensatory mechanisms and plasticity in patients with functional deficits or at high risk for tumor-related affection of language function [28,42,43]. However, this study aimed at evaluating a practicable way to use nTMS mapping and related nTMS-based DTI FT for risk evaluation, thus not setting the focus on distinct assessment of plastic effects that can occur as a result of the tumor. The results show success in this regard, but future insights into the language network may require update of the approach for tract selection and measurements. Third, we only applied the approach of nTMS-based DTI FT in this study, but did not perform comparisons to tractography using seeding based on anatomical landmarks. In this context, a previous study emphasized that more language-related tracts can be visualized when using the nTMS-based approach compared with seeding using predefined anatomical landmarks, but for the AF, anatomy-based seeding was superior by allowing tract delineation in more patients [44]. In the present study, we took advantage of the nTMS-based approach as we aimed at tractography of various language-related tracts. Furthermore, tracking of the AF was possible in all enrolled patients in the present study, as opposed to the previous investigation [44]. Fourth, we used deterministic tractography based on DTI sequences as commonly performed in the routine neurosurgical setting. However, it has repeatedly been shown that the DTI technique may be prone to errors, particularly in the situation of crossing or kissing fibers or presence of peritumoral edema, among other influencing factors [45,46]. Other sequences and tracking algorithms are developed to become applicable in the clinical setting, yet alternatives to conventional DTI and related analyses are still elaborate and mostly far from routine use [47,48]. Thus, with technical refinements, measurements of LTDs that are even closer to reality may soon become possible.

## 5. Conclusions

This is the largest study evaluating the application of both preoperative functional mapping and function-based tractography for motor and language function regarding risk stratification in patients suffering from functionally eloquent tumors in a real-world clinical setting. The results provide a feasible tool for neurooncologists, neurosurgeons, tumor boards, and potentially radiation oncologists to provide individual risk assessment for each patient considering large cohort data on the one hand, as well as individual assessment on the other.

## Figures and Tables

**Table 1:** Patient details.

Item			Motor Mappings	Language Mappings
Number of Mappings (N)			150	100
Number of Patients (N)			118	98
Age			58.2 ± 15.2 [21.1–89.7]	55.6 ± 15.6 [18.9–82.7]
(in years, mean ± SD [range])				
Gender			64.4/35.6	52.0/48.0
(in %, male/female)				
Maximum Follow-Up			10.8 ± 6.5 [3.0–28.4]	8.8 ± 5.6 [3.0–33.7]
(in months, mean ± SD [range])				
Affected Hemisphere			39.0/61.0	100.0/0.0
(in %, left/right)				
Awake Surgery (in %)			0.0	21.0
Type of Surgery			96.0/4.0	96.0/4.0
(in %, resection/biopsy)				
**Tumor Entity** (in %)	Glioma WHO grade I		0.0	5.1
	Glioma WHO grade II		0.0	4.1
	Glioma WHO grade III		21.2	14.3
	Glioma WHO grade IV		78.8	49.0
	Arteriovenous malformation		0.0	4.1
	Cavernoma		0.0	5.1
	Metastasis		0.0	18.4
**IDH Status** (in % for patients with glioma, IDH-wildtype/IDH-mutant)			71.2/28.8	71.8/28.2
**Functional Deficits** (in %)	Preoperative	*No*	69.3	58.0
		*Yes*	30.7	42.0
	Postoperative	*No*	56.0	38.0
		*Yes*	44.0	62.0
	Follow-up	*No*	58.7	57.0
		*Yes*	41.3	43.0
	Surgery-related	*None*	76.0	62.0
		*Transient*	2.0	17.0
		*Permanent*	22.0	21.0
	Perioperative ischemia (in patients with surgery-related transient or permanent deficits)		44.4	57.9

This table provides an overview of patient-related characteristics. Information on the tumor entity is based on histopathological evaluation and grading of the World Health Organization (WHO), including data on the status of mutation of the isocitrate dehydrogenase (IDH) in glioma patients. Values are given as absolute or relative frequencies or as means ± standard deviation (SD) and ranges.

**Table 2:** Mapping and tractography details.

Motor Mappings and nTMS-Based Tractography			
Resting Motor Threshold	Unaffected hemisphere	33.5 ± 8.2 [21.0–56.0]	*p* = 0.1719
(in % of stimulator output, mean ± SD [range])	Tumor-affected hemisphere	35.3 ± 10.1 [19.0–99.0]	
100% Fractional Anisotropy Threshold (mean ± SD [range])		0.34 ± 0.07 [0.21–0.57]	
**Language Mappings and nTMS-Based Tractography**			
Resting Motor Threshold	Unaffected hemisphere	35.6 ± 6.9 [22.0–55.0]	*p* = 0.0012
(in % of stimulator output, mean ± SD [range])	Tumor-affected hemisphere	36.4 ± 9.1 [21.0–75.0]	
100% Fractional Anisotropy Threshold (mean ± SD [range])		0.28 ± 0.06 [0.15–0.47]	
AF is Closest Tract to Tumor (in %)		27.0	
Other Closest Language-Related Tract (in %)	SLF	15.0	
	ILF	32.0	
	FoF	52.0	
	UC	1.0	

This table gives an overview of mapping and tractography characteristics including information on the fractional anisotropy threshold (FAT) used for tracking of the corticospinal tract (CST), arcuate fascicle (AF), or other closest language-related tract considering the superior longitudinal fascicle (SLF), inferior longitudinal fascicle (ILF), uncinate fascicle (UC), and frontooccipital fascicle (FoF). Values are given as relative frequencies or as means ± standard deviation (SD) and ranges.

**Table 3:** Lesion-to-tract distances (LTDs) for surgery-related motor deficits.

LTD—CST (in mm)		25% FAT		50% FAT		75% FAT	
		Mean ± SD [Range]	*p* (vs. None/Transient Deficits)	Mean ± SD [Range]	*p* (vs. None/Transient Deficits)	Mean ± SD [Range]	*p* (vs. None/Transient Deficits)
**Surgery-Related Motor Deficits**	None/transient	10.0 ± 9.9 [0.0–42.4]	-	12.7 ± 10.8 [0.0–48.2]	-	14.9 ± 10.8 [0.0–48.2]	-
	Permanent (all patients)	2.0 ± 3.3 [0.0–12.2]	<0.0001	3.1 ± 3.6 [0.0–12.4]	<0.0001	4.3 ± 4.1 [0.0–13.4]	<0.0001
	Permanent (excluding patients with perioperative ischemia)	0.6 ± 1.1 [0.0–3.0]	<0.0001	1.8 ± 2.2 [0.0–5.5]	<0.0001	3.1 ± 3.4 [0.0–11.8]	<0.0001

This table provides the results regarding LTDs in relation to the corticospinal tract (CST), which were compared between patients presenting with no or transient and permanent surgery-related paresis. For diffusion tensor imaging fiber tracking (DTI FT) based on navigated transcranial magnetic stimulation (nTMS), three different adjustments for the fractional anisotropy (FA) were used, which were 25%, 50%, and 75% of the individual FA threshold (FAT). The cells for LTDs depict the means ± standard deviation (SD) and ranges.

**Table 4:** Lesion-to-tract distances (LTDs) for surgery-related language deficits.

LTD—AF (in mm)		25% FAT		50% FAT		75% FAT	
		Mean ± SD [Range]	*p* (vs. None/Transient Deficits)	Mean ± SD [Range]	*p* (vs. None/Transient Deficits)	Mean ± SD [Range]	*p* (vs. None/Transient Deficits)
**Surgery-Related Language Deficits**	None/transient	12.2 ± 11.5 [0.0–41.1]	-	16.0 ± 13.2 [0.0–57.7]	-	18.4 ± 13.7 [0.0–58.3]	-
	Permanent (all patients)	2.8 ± 6.8 [0.0–24.6]	0.0005	4.4 ± 8.7 [0.0–25.8]	0.0012	8.3 ± 10.0 [0.0–27.1]	0.0491
	Permanent (excluding patients with perioperative ischemia)	1.0 ± 2.2 [0.0–4.9]	0.0119	4.7 ± 6.3 [0.0–13.3]	0.0579	7.8 ± 5.8 [1.3–15.4]	0.1095
**LTD—Other Closest Language-Related Tract (in mm)**		**25% FAT**		**50% FAT**		**75% FAT**	
		**Mean ± SD [Range]**	***p* (vs. None/Transient Deficits)**	**Mean ± SD [Range]**	***p* (vs. None/Transient Deficits)**	**Mean ± SD [Range]**	***p* (vs. None/Transient Deficits)**
**Surgery-Related Language Deficits**	None/transient	10.3 ± 13.0 [0.0–53.0]	-	13.0 ± 13.3 [0.0–54.2]	-	16.1 ± 14.5 [0.0–54.7]	-
	Permanent (all patients)	3.4 ± 5.9 [0.0–23.1]	0.0113	5.2 ± 6.7 [0.0–23.9]	0.0147	7.6 ± 7.4 [0.0–24.1]	0.0435
	Permanent (excluding patients with perioperative ischemia)	6.2 ± 8.1 [0.0–23.1]	0.4179	7.6 ± 8.2 [0.0–23.9]	0.2829	9.4 ± 8.5 [0.0–24.1]	0.2299

This table provides the results regarding LTDs in relation to the arcuate fascicle (AF) and other closest language-related tracts, which were compared between patients presenting with no or transient and permanent surgery-related aphasia. For diffusion tensor imaging fiber tracking (DTI FT) based on navigated transcranial magnetic stimulation (nTMS), three different adjustments for the fractional anisotropy (FA) were used, which were 25%, 50%, and 75% of the individual FA threshold (FAT). The cells for LTDs depict the means ± standard deviation (SD) and ranges.

**Table 5:** Correlations between lesion-to-tract distances (LTDs) and surgery-related motor or language deficits.

Correlations		Surgery-Related Deficits (All Patients)			Surgery-Related Deficits (Excluding Patients with Perioperative Ischemia)		
		25% FAT	50% FAT	75% FAT	25% FAT	50% FAT	75% FAT
LTD—CST	r	−0.3933	−0.4136	−0.4473	−0.3910	−0.3923	−0.3994
	*p*	<0.0001	<0.0001	<0.0001	<0.0001	<0.0001	<0.0001
LTD—AF	r	−0.3590	−0.3775	−0.2888	−0.2918	−0.2662	−0.2592
	*p*	0.0006	0.0014	0.0490	0.0135	0.0473	0.1111
LTD—Other Closest Language-Related Tract	r	−0.2525	−0.2551	−0.2622	−0.0853	−0.1168	−0.1683
	*p*	0.0112	0.0147	0.0430	0.4575	0.3215	0.2478

This table depicts the Spearman correlation coefficients and related *p*-values for associations between the status of surgery-related deficits (no or transient and permanent surgery-related paresis or aphasia) and LTDs, considering the corticospinal tract (CST), arcuate fascicle (AF), and other closest language-related tracts.

## Abbreviations

3D	Three-dimensional
AF	Arcuate fascicle
BMRC	British Medical Research Council
CST	Corticospinal tract
DES	Direct electrical stimulation
DTI	Diffusion tensor imaging
DTI FT	Diffusion tensor imaging fiber tracking
FA	Fractional anisotropy
FAT	Fractional anisotropy threshold
FL	Fiber length
FLAIR	Fluid attenuated inversion recovery
FoF	Frontooccipital fascicle
FU	Follow-up
IDH	Isocitrate dehydrogenase
ILF	Inferior longitudinal fascicle
LAD	Lesion-to-activation distance
LTD	Lesion-to-tract distance
MEP	Motor evoked potential
MRI	Magnetic resonance imaging
nTMS	Navigated transcranial magnetic stimulation
rMT	Resting motor threshold
ROI	Region of interest
SD	Standard deviation
SLF	Superior longitudinal fascicle
TE	Echo time
TR	Repetition time
UC	Uncinate fascicle
WHO	World Health Organization
